# The Acceptability of Digital Technology and Tele-Exercise in the Age of COVID-19: Cross-sectional Study

**DOI:** 10.2196/33165

**Published:** 2022-04-13

**Authors:** Vanda Ho, Reshma A Merchant

**Affiliations:** 1 Division of Geriatric Medicine Department of Medicine National University Hospital Singapore Singapore

**Keywords:** senior, telehealth, digital exercise, acceptability, telemedicine, elderly, older adults, outcome, isolation, decline, function, adoption, perception, exercise, physical activity, questionnaire, COVID-19

## Abstract

**Background:**

With the COVID-19 pandemic, telehealth has been increasingly used to offset the negative outcomes of social isolation and functional decline in older adults. Crucial to the success of telehealth is end user adoption.

**Objective:**

This study aims to investigate perception and acceptability of digital technology among Asian older adults.

**Methods:**

The Healthy Ageing Promotion Program for You (HAPPY) dual-task exercise was conducted virtually to participants aged ≥60 years. Questionnaires were administered digitally and collected data on demographics, perceptions of digital technology and evaluation of HAPPY, the 6-item Lubben Social Network Scale, intrinsic capacity using the Integrated Care for Older People tool, and a functional screening with the FRAIL scale and five chair rises. Descriptive analysis was used.

**Results:**

A total of 42 participants were digitally interviewed. The mean age was 69.1 (4.7) years. Hearing, vision, and 3-item recall difficulty were present in 14% (n=6), 12% (n=5), and 24% (n=10) of participants, respectively. Of the participants, 29% (n=12) had possible sarcopenia and 14% (n=6) were prefrail. Around 24% (n=10) were at risk of social isolation. Most of the participants (n=38, 91%) agreed that technology is good, and 79% (n=33) agreed that technology would allow them to be independent for longer. Over three-quarters of participants (n=33, 79%) agreed that they have the necessary knowledge, and 91% (n=38) had technological assistance available. However, 57% (n=24) were still apprehensive about using technology. Despite 71% (n=30) of older adults owning their devices, 36% (n=15) felt finances were limiting. Through digital HAPPY, 45% (n=19) of participants reported feeling stronger, 48% (n=20) had improved spirits, and 40% (n=17) and 38% (n=16) had improved mood and memory, respectively.

**Conclusions:**

The majority of older adults in this study believed in digital technology and had the necessary knowledge and help, but almost half still felt apprehensive and had financial barriers to adopting technology. A digitally administered exercise program especially in a group setting is a feasible option to enhance intrinsic capacity in older adults. However, more work is needed in elucidating sources of apprehension and financial barriers to adopting technology.

## Introduction

State-mandated lockdowns during the COVID-19 pandemic, while necessary to curb spread, has led to social isolation, reduced physical activity, and functional decline [1]. To mitigate the effects of the pandemic, telehealth has been used in many countries to offset the negative outcomes, especially among vulnerable older adults who are often advised to stay at home [2]. There are many uses of telehealth, such as screening for geriatric syndromes, monitoring, management, and psychosocial support. Telehealth has also been used to deliver physical exercise and social activities to encourage physical activity and reduce loneliness [3,4].

The success of telehealth depends on end user adoption. Age is often quoted as a barrier to accepting new technology. Several themes and predictors have been identified in the perception and readiness of using digital technology including self-efficacy, digital literacy, obstacles to using technology, prior experience, frequency of use, sources of support, performance expectancy, perceived usefulness, social influence, computer anxiety, perceived security, and physician’s opinion [5,6]. Several suggestions have been made to target these factors, such as reimbursement change, provision of telecommunication devices as a medical necessity, and improving accessibility [7].

**Figure 1:**
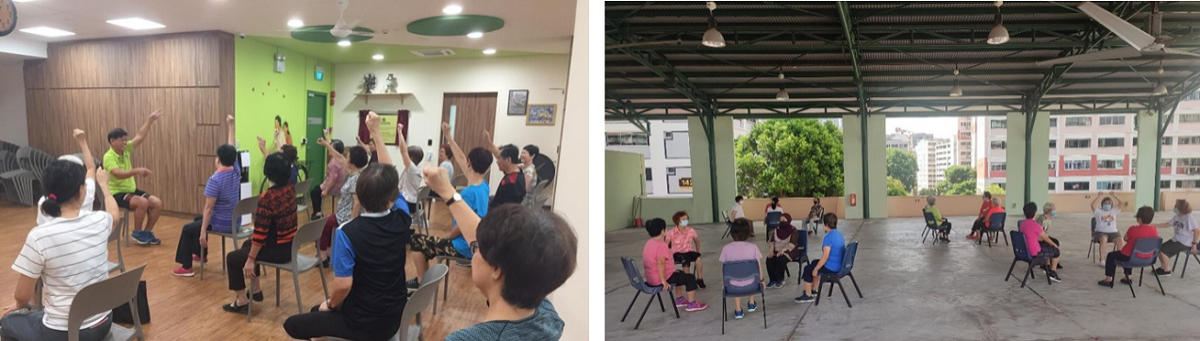
The Healthy Ageing Promotion Program for You (HAPPY) conducted on-site at community centers pre–COVID-19 (left) and in outdoor spaces with social distancing post–COVID-19 (right).

Our paper describes technology acceptability in older adults who were undergoing an on-site frailty intervention that subsequently transited into digital form. The Healthy Ageing Promotion Program for You (HAPPY) dual-task exercise program was first rolled out in 2017. The program was adapted from Cognicise and is a group multicomponent exercise focusing on cognition and physical function, originating from the National Center for Geriatrics and Gerontology in Nagoya, Japan. Prior to the COVID-19 pandemic, this program was ongoing in more than 50 different sites with at least 700 participants across Singapore and has shown to reverse frailty and improve cognition [8] (Figure 1).

**Figure 2:**
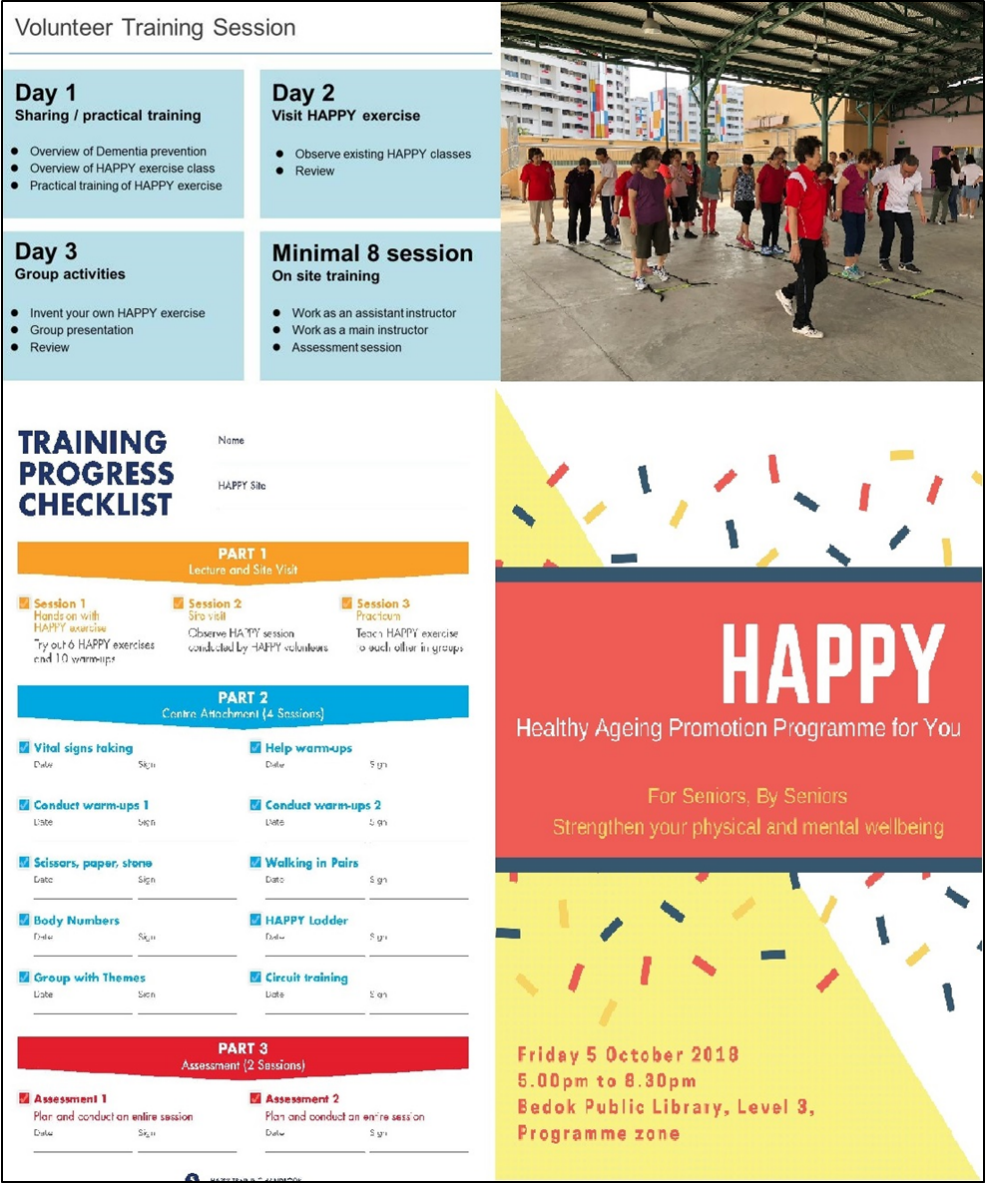
Training program. HAPPY: Healthy Ageing Promotion Program for You.

On-site HAPPY was conducted by health coaches and peer leaders who were trained to perform dual-task exercise by Japanese physiotherapists. The training program consisted of 3 sessions of theoretical training and cocreation of dual-task exercise and 8 hours of on-site training as assistant instructor and assessment (Figure 2). Digital HAPPY in early stages was led only by health coaches.

**Figure 3:**
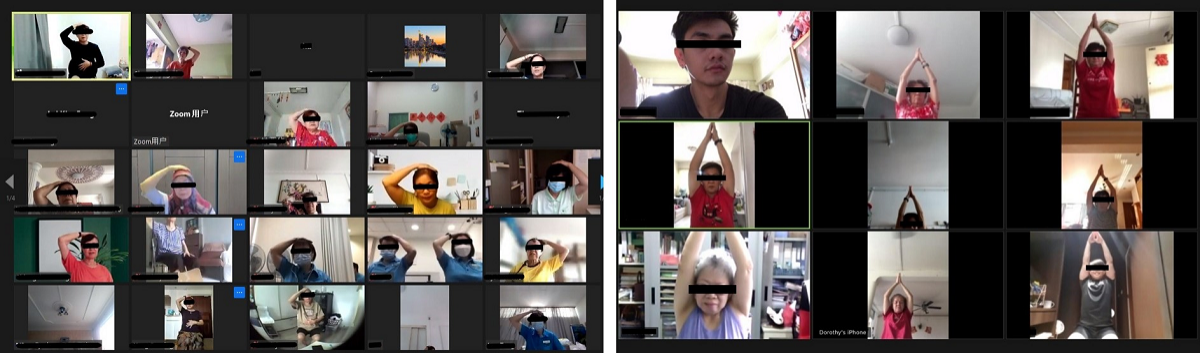
Digital Healthy Ageing Promotion Program for You (HAPPY) administered over the Zoom platform. Initial roll out had several issues with the inability to turn on the camera and not knowing how to angle the camera toward themselves (left). Over the year, participants acquired skills on using Zoom (right).

During the COVID-19 lockdown, the program was delivered virtually through the Zoom platform. More than 100 participants including their peers continued to participate. In the early phase of the national lockdown in April 2020, about 40% of participants agreed to participate in digital HAPPY, but only 14% eventually participated. As the nation remained in a semilockdown state, efforts to encourage more older adults to join the virtual platform persisted. Since March 2021, more than 700 participants now participate in digital HAPPY (Figure 3).

Initial recruitment and evaluation were focused on those who were prefrail or had underlying cognitive impairment, but anyone in the community could join in the program [8]. An invitation to participate in digital HAPPY was sent through a WhatsApp message when activities were discontinued overnight and all older adults were advised to stay at home. Participation in digital HAPPY was open to anybody who was interested, was comfortable in using the Zoom platform, and owned a personal device.

Little is known on the perception and acceptability of digital technology among Asian older adults, which is critical in increasing the uptake of telehealth. In addition, due to decline in sensory input and mobility with aging, it is less clear if tele-exercise has a perceived positive impact on older adults. Hence, as our frailty intervention underwent transition from on-site to digital, it was timely for us to study the acceptability of technology and perceived self-reported benefit of tele-exercise in our community-dwelling older adults during the pandemic lockdown.

## Methods

This is a cross-sectional descriptive study on technology acceptability in older adults, nested as part of a larger study—the HAPPY program previously described.

### Recruitment

All existing participants of digital HAPPY aged ≥60 years were invited via WhatsApp to participate in the digital survey regarding technology acceptability between September and December 2020. Positive responders were sent the weblink. Consent was taken online. Participants needed to have access to technology, defined as older adults who had devices (eg, mobile, tablet, or computer), internet connection, and ability to use WhatsApp and Zoom.

The questionnaire can be found online [9], including questions on demographics, health conditions, lifestyle habits, acceptability of digital technology, and perceived self-reported benefits of HAPPY, and were administered by a trained research assistant. Social isolation was measured using the 6-item Lubben Social Network Scale [10]. A score below 12 (maximum 30) suggests a risk of social isolation. Intrinsic capacity was measured using the Integrated Care for Older People tool administered digitally, which included questions on cognition, psychology, and vitality, and a hearing and vision screening [11]. Participants were also asked to complete five chair rises. The cutoff for possible sarcopenia was based on the Asian Workgroup for Sarcopenia 2019 recommendations of ≥12 seconds [12]. The FRAIL scale was used to screen for frailty where scores of 3 to 5 represent frail and 1 to 2 represent prefrail [13].

### Statistical Analysis

As this was an exploratory descriptive study, no sample size calculation was performed. Due to the small sample size, descriptive analysis was conducted.

### Ethics Approval

Ethics approval was obtained from the National Healthcare Group Domain Specific Review Board (reference number: 2020/00668). Reporting is in accordance with the STROBE (Strengthening the Reporting of Observational Studies in Epidemiology) checklist.

## Results

Only 42 (14%) of the participants who participated in the first 3 months agreed to be interviewed digitally, as many were apprehensive about digital consenting. The mean age was 69.1 (SD 4.7) years; 93% (n=39) of participants were female and 29% (n=12) of participants had up to 6 years of education, with 91% (n=38) being from the Chinese ethnic group. Only 7% (n=3) were employed, 62% (n=26) were retired, and 55% (n=23) had two or more chronic illness. Hearing, vision, and the 3-item recall difficulty was present in 15% (n=6), 12% (n=5), and 24% (n=10), respectively. Functionally, 29% (n=12) had possible sarcopenia, 15% (n=6) were prefrail, and 19% (n=8) had 1 or more falls in the past year. Slightly more than 1 in 5 were at risk of social isolation. Of the social media platforms, 93% (n=39) used WhatsApp, 79% (n=33) used YouTube, 21% (n=9) used WeChat, 19% (n=8) used Telegram, and 12% (n=5) used Instagram. Only 71% (n=30) owned their own device.

On the acceptability of technology (Table 1), 91% (n=38) agreed that technology is a good idea and 79% (n=33) agreed that technology would allow them to be independent for longer. Someone was available for technological assistance for 91% (n=38) of participants. The majority (n=36, 86%) had access to technology. Financial status did not limit their activities in using technology in 45% (n=19) of participants. Despite that 79% (n=33) agreed that they have the necessary knowledge to use the system and 86% (n=36) agreed that they could complete the task if someone showed them or through an instruction manual, 57% (n=24) were apprehensive about using technology. In addition, 41% (n=17) disagreed or were neutral that technology is easy to use and that technology is easy to learn.

**Figure 4:**
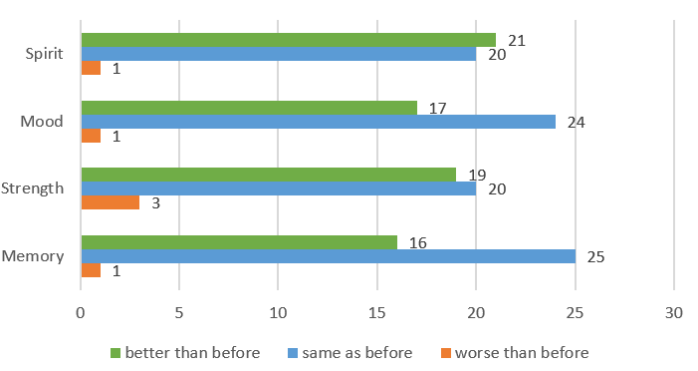
Bar Chart of Characteristics Before and After Online HAPPY Intervention. HAPPY: Healthy Ageing Promotion Program for You.

Of the participants, 93% (n=39) had attended on-site HAPPY exercises before. With digital HAPPY, 45% (n=19) reported feeling stronger than before, 48% (n=20) reported improvement in spirits, 40% (n=17) improvement in mood, and 38% (n=16) reported improvement in memory (Figure 4).

**Table 1:** Acceptability of technology in older adults (N=42).

	Strongly disagree, n (%)	Disagree, n (%)	Slightly disagree, n (%)	Neutral, n (%)	Slightly agree, n (%)	Agree, n (%)	Strongly agree, n (%)
You have the knowledge necessary to use the system	0 (0)	6 (14)	0 (0)	3 (7)	10 (24)	20 (48)	3 (7)
A specific person (or group) is available for assistance with technology difficulties	0 (0)	2 (5)	0 (0)	2 (5)	1 (2)	36 (86)	1 (2)
Your financial status does not limit your activities in using technology	0 (0)	15 (36)	0 (0)	8 (19)	1 (2)	17 (41)	1 (2)
When you want or need to use technologies, they are accessible for you	0 (0)	4 (10)	1 (2)	1 (2)	1 (2)	34 (81)	1 (2)
Your family and friends think/support that you should use technology	0 (0)	1 (2)	0 (0)	7 (17)	1 (2)	32 (76)	1 (2)
Using technology is a good idea	0 (0)	2 (5)	0 (0)	2 (5)	3 (7)	24 (57)	11 (26)
Using technology would allow you to be independent for longer	1 (2)	4 (10)	0 (0)	4 (10)	3 (7)	24 (57)	6 (14)
Technology is easy to use	0 (0)	5 (12)	5 (12)	7 (17)	7 (17)	17 (41)	1 (2)
Technology is easy to learn	0 (0)	6 (14)	4 (10)	7 (17)	10 (24)	15 (36)	0 (0)
You could complete a task using technology if there is someone to demonstrate how	1 (2)	3 (7)	1 (2)	1 (2)	10 (24)	21 (50)	5 (12)
You could complete a task using technology if you have just the instruction manual for assistance	1 (2)	1 (2)	2 (5)	2 (5)	12 (29)	22 (52)	2 (5)
You feel apprehensive about using the technology	0 (0)	13 (31)	1 (2)	4 (10)	10 (24)	12 (29)	2 (5)

## Discussion

### Principal Findings

Our study is particularly relevant during the current COVID-19 pandemic, with the reinforcement of social distancing especially among older adults. More than three-quarters of the seniors had the necessary knowledge to use the system. COVID-19 has accelerated technology adoption among older adults not only in the health care sector but also on social platforms, education, grocery and food delivery, socialization, and tele-exercise. Almost all the older adults used WhatsApp and more than three-quarters used YouTube.

Odeh and colleagues [14] found that older adults were generally satisfied with equipment use and not concerned about confidentiality or absence of direct contact with health care, and telehealth increased their self-efficacy in managing their own health. Results of our survey indicate that more than three-quarters of older adults acknowledged the potential of technology in keeping them independent for longer. Help from family and friends was also available when needed, which may reflect the benefits of living in multigenerational homes typical of an Asian society.

While the majority had access to technology, finance was an issue for more than half. The digital divide can be affected at four different levels including motivational access, material access, skills access, and use access [15]. Nearly half of our participants were neutral or disagreed that technology is easy to use and learn, with more than half of participants feeling apprehensive about technology. Hearing, vision, or cognitive impairment could have contributed to the difficulties. Further studies are needed to identify sources of these apprehension to address them. Navigating the complexity of technology, such as purchasing and learning to use the equipment or app and connecting to the internet, requires upskilling of the older adults, as well as providing them with financial resources. Singapore launched the Seniors Go Digital initiative in May 2020 and has made the phone plans and equipment more affordable for older adults to reduce the digital divide [16]. There has been workflows instituted since the advent of COVID-19 to facilitate this [17], but policies continue to evolve to make health care and services more accessible.

There has been emerging research on digitally administered social activities and exercise for improving strength and reducing falls and loneliness, and studies have shown that uptake was influenced by perceived usefulness, enjoyment, social influence, gender, experience, and ease of use among other factors [18]. One in two older adults are at risk of social isolation locally but only one in four of our participants were at risk [19]. Many of them were already participating in HAPPY exercises before going digital and were socially connected prior to the lockdown. Older adults reported feeling stronger, better spirit, and improved mood and memory after participating in digital HAPPY. Improvement in mental and physical health have been shown with physical exercise, but the benefits seen and attained with tele-exercise during the pandemic lockdown is even more crucial to reduce functional decline and loneliness. We have previously found that these activities provide windows of opportunities used to digitally assess sarcopenia risk, suggesting that both screening and intervention can be performed on the same platform [20]. Hence, tele-exercise helped improve access and ensure they remained connected with the wide community even during social distancing.

To summarize, our study indicates that older adults are more than happy to adopt digital technology, and tele-exercise can feasibly be used to prevent and delay functional decline and loneliness. More work needs to be done on improving electronic interfaces that can be user friendly to older adults who have multiple sensory impairments and joint disability. While these efforts in reducing the digital divide and improving access to telehealth and digital technology are still underway, in certain population groups such as those with sensory or cognitive impairment, in-person clinic or home visits would still be the mainstay.

The majority of older adults believe in digital technology and have the necessary knowledge and help, but almost half still feel apprehensive and have financial barriers to adopting technology. A digitally administered exercise program especially in a group setting is a feasible option to improve function.

### Limitations

Our participants are not representative of a community sample, as they were preselected themselves to participate in digital HAPPY, and those who agreed for the digital interview already had the necessary knowledge. Despite that, more than half of the participants were apprehensive and cited financial barriers to access technology. While our study population is small, it did show that tele-exercise is beneficial in improving physical and cognitive function. Further population studies are needed to identify the areas of apprehension to and benefits of initiatives and taking up Seniors Go Digital.

### Conclusions

Digital technology including telehealth is increasingly important in this COVID-19 era. Older adults are generally accepting of technology, with top barriers being technology specific, and they are willing to adopt and adapt. However, more studies are needed to elucidate the source of their apprehension and to guide interventions to boost telemedicine infrastructure. Tele-exercise has the potential to be a useful modality to reduce functional decline and social isolation in older adults.

## Abbreviations

HAPPY: Healthy Ageing Promotion Program for You
STROBE: Strengthening the Reporting of Observational Studies in Epidemiology
